# Enzyme-Induced Transition of the Morphology of Polyelectrolyte Complexes

**DOI:** 10.1021/acs.biomac.6c00029

**Published:** 2026-02-13

**Authors:** Chaeyoung Lim, Whitney C. Blocher McTigue

**Affiliations:** Department of Chemical and Biomolecular Engineering, 1687 Lehigh University, Bethlehem, Pennsylvania 18015, United States

## Abstract

Polyelectrolyte complexes (PECs) can exist as liquid coacervates or solid precipitates with triggers such as salt, pH, or temperature driving transitions. However, it is unclear how enzymatic chain scission reorganizes solid PECs and alters their mechanics. We probed cellulase-mediated remodeling of solid PECs formed from carboxymethyl cellulose (CMC) and short or long poly­(diallyldimethylammonium chloride) (PDADMAC). Turbidity and microscopy showed that short PDADMAC/CMC complexes decreased in turbidity and became liquid-like as enzyme dose and time increased. In contrast, long PDADMAC complexes exhibited a transient turbidity increase at high cellulase concentration and formed droplet-like phases consistent with competing PDADMAC/cellulase coacervation. Zeta potential supported cellulase binding to PDADMAC, which reduced the positive charge available for CMC bridging. Rheology showed dose- and time-dependent softening, while long PDADMAC remained more elastic and relaxed more slowly. Thus, the enzyme dose and polycation length jointly control the kinetics of coupled structural and mechanical transitions in solid PECs.

## Graphical abstract

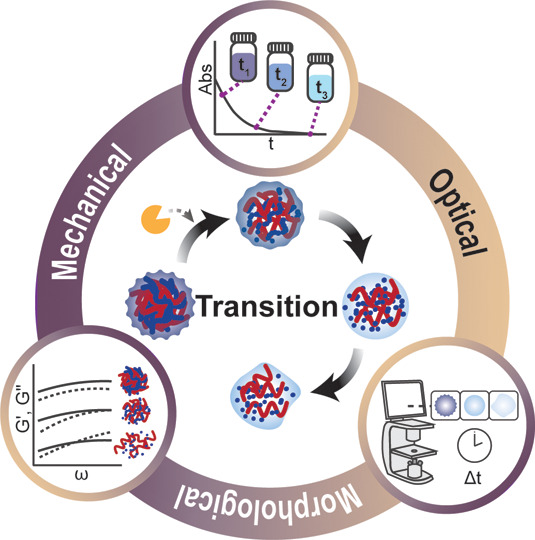

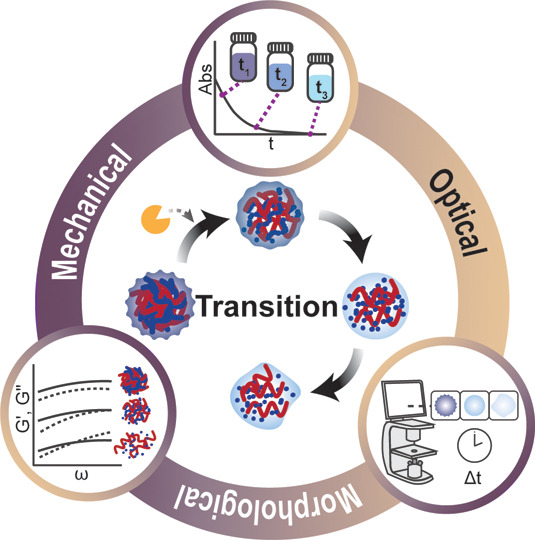

## 1 Introduction

When mixed in aqueous solution, oppositely charged polyelectrolytes phase-separate into coexisting polymer-poor and polymer-rich phases, known as polyelectrolyte complexes (PECs).^1–3^ This phase transition can produce either liquid–solid or liquid–liquid coexistence and is driven by electrostatic interactions between oppositely charged chains, along with the entropic gain from counterion release.^4,5^ Consequently, PECs span a continuum of physical states, ranging from dynamic coacervates to kinetically trapped, stable solid precipitates.^6–10^ While liquid coacervates have been widely studied due to their favorable processability and utility in applications,^11–15^ solid precipitates remain comparatively less explored, despite still exhibiting a range of physical states such as glassy, gel-like, or partially relaxed states with tunable phase behavior and mechanics.^12^

Solid PECs are structurally stable because strong ionic interactions and relatively low water content give rise to a rigid, glassy state.^7,16^ This stability makes them suitable for applications requiring long-term structural integrity and mechanical stability.^17,18^ At the same time, several studies have shown that these solid precipitates can be relaxed under appropriate external stimuli.^7,19–23^ For example, increasing salt concentration can continuously drive solid precipitates to coacervates and, eventually, to homogeneous solutions.^19,23^ These salt-induced transitions have been characterized by tracking *G*′ and *G*″, along with changes in phase morphology, using rheology and microscopy.^7,24–26^ The authors used salt and temperature as time-salt or time–temperature superposition variables to map the relaxation dynamics of PECs across the solid-to-liquid continuum. These stimulus-responsive studies demonstrate that solid PECs are not inherently rigid but can exhibit a broad range of viscoelastic responses when their ionic environment is altered. However, they primarily rely on bulk changes in the surrounding medium and do not directly address how more localized triggers reorganize the dense phase.

Selectively degradable bonds and enzymes have been used to access different mechanisms of responsiveness in PECs. Early work by Leclercq and co-workers involved the design of biomimetic PECs in which selective enzymatic chain scission served as a tool to probe complex behavior. Using trypsin, they selectively degraded poly­(l-lysine) (PLL) within complexes while leaving nondegradable polyanions intact, enabling PEC disassembly and recovery of the polyanion for analysis.^27,28^ Insua and co-workers reported enzyme-responsive polyion complex (PIC) nanoparticles in which an anionic enzyme-cleavable block was paired with cationic antimicrobial polymers, allowing pathogenic elastase to trigger particle destabilization and enhance local antimicrobial activity.^29^ More recently, Li et al. developed phosphatase-responsive polyelectrolyte complexes for the delivery of α-helical antimicrobial polypeptides, where dephosphorylation of an anionic block modulates complex stability, peptide conformation, and antibacterial efficacy.^30^ Protease-driven phase separation has also been demonstrated in elastin-like polypeptides, where inserting a protease cleavage site into an amphiphilic elastin-like polypeptide leads to time-dependent changes in turbidity and the emergence of liquid droplets upon proteolysis.^31^ These studies show that breaking covalent bonds in one component provides a robust handle for manipulating self-assembled phases. However, most enzyme-responsive PEC and PIC systems have focused on nanoscale micelles or liquid coacervates in dispersion and have primarily emphasized cargo release or activity rather than directly following how enzymatic degradation of a single component reorganizes the dense phase of preformed solid complexes and alters their viscoelastic response.

In this study, we investigate whether solid complexes formed from mixtures of poly­(diallyldimethylammonium chloride) (PDADMAC) and carboxymethyl cellulose (CMC) can be structurally and mechanically remodeled through enzymatic cleavage of CMC. We prepared solid PDADMAC/CMC precipitates and triggered their evolution by adding cellulase at varying concentrations, with PDADMAC serving as a nondegradable polycation and CMC as a cellulase-degradable polyanion. We tracked the time-dependent structural response using turbidity kinetics and bright-field microscopy to monitor the transition from densely packed precipitates to more liquid-like materials. We then used ζ-potential measurements to probe the electrostatic interaction between cellulase and PDADMAC, which could influence the complex reorganization. Finally, we used rheology to link these morphological changes to the time-dependent evolution of the linear viscoelasticity. Overall, this framework directly links enzymatic chain scission within a solid PEC to coupled structural and mechanical transitions.

## 2 Experimental Section

### 2.1 Materials

Low molecular weight poly­(diallyldimethylammonium chloride) (PDADMAC, average *M*_w_ ∼8500 g/mol, 28 wt % in H_2_O) was purchased from Polysciences, and a higher molecular weight PDADMAC (average *M*_w_ < 100,000 g/mol, 35 wt % in H_2_O) was purchased from Sigma-Aldrich. Short-length PDADMAC (*M*_w_ ∼8500) and long-length PDADMAC with *M*_w_ < 100,000 g/mol are referred to as sPDADMAC and lPDADMAC, respectively. Carboxymethyl cellulose (CMC, degree of substitution (DS) 0.6, degree of polymerization (DP) 1050) and 2-[4-(2-hydroxyethyl)­piperazineethanesulfonic acid] (HEPES) were purchased from Thermo Fisher Scientific. Cellulase, produced by a fungal strain of *Aspergillus niger*, was purchased from TCI Chemicals. All materials were used as received. 10 mM stock solutions of both PDADMACs, CMC, and cellulase were prepared on a monomer basis. A 0.5 M HEPES buffer solution was also prepared. The pH of each solution was adjusted to 7.0 using 0.1 or 1 M hydrochloric acid and sodium hydroxide. All stock solutions were stored at 4 °C after preparation.

### 2.2 Polyelectrolyte Complexation

All samples were prepared at room temperature in 1.5 mL microcentrifuge tubes (Eppendorf) using deionized water (Aries FilterWorks, 18.2 MΩ·cm) containing 10 mM HEPES buffer at pH 7.0. Polyelectrolyte complexes were formed by mixing defined volumes of CMC with either short-chain (sPDADMAC) or long-chain PDADMAC (lPDADMAC), as described in Table S1 to achieve a 3 mM total polymer concentration on a monomer basis. The resulting mixtures were transferred to a 384-well plate for turbidity measurements and brightfield microscopy.

### 2.3 Cellulase-Induced Phase Transition

This study examined the effects of the enzyme concentration on the solid-to-liquid phase transition of PDADMAC/CMC complexes. Samples were prepared at *x*_PDADMAC_ = 0.475 (peak in Figure 1b) and total polymer concentration was 3 mM, in 10 mM HEPES buffer at pH 7.0. Table S2 summarizes the composition in which various concentrations of cellulase (0, 0.02, 0.04, 0.08, 0.2, 0.4, and 0.8 mM) were added to preformed PDADMAC/CMC complexes prepared at 3 mM total polymer concentration. After preparation, the samples were transferred into a 96-well plate for turbidity measurements taken over 72 h. Samples with varying enzyme concentrations were used to investigate how cellulase affects the solid-to-liquid phase transition of PDADMAC/CMC complexes at a total polymer concentration of 3 mM.

**Figure 1:**
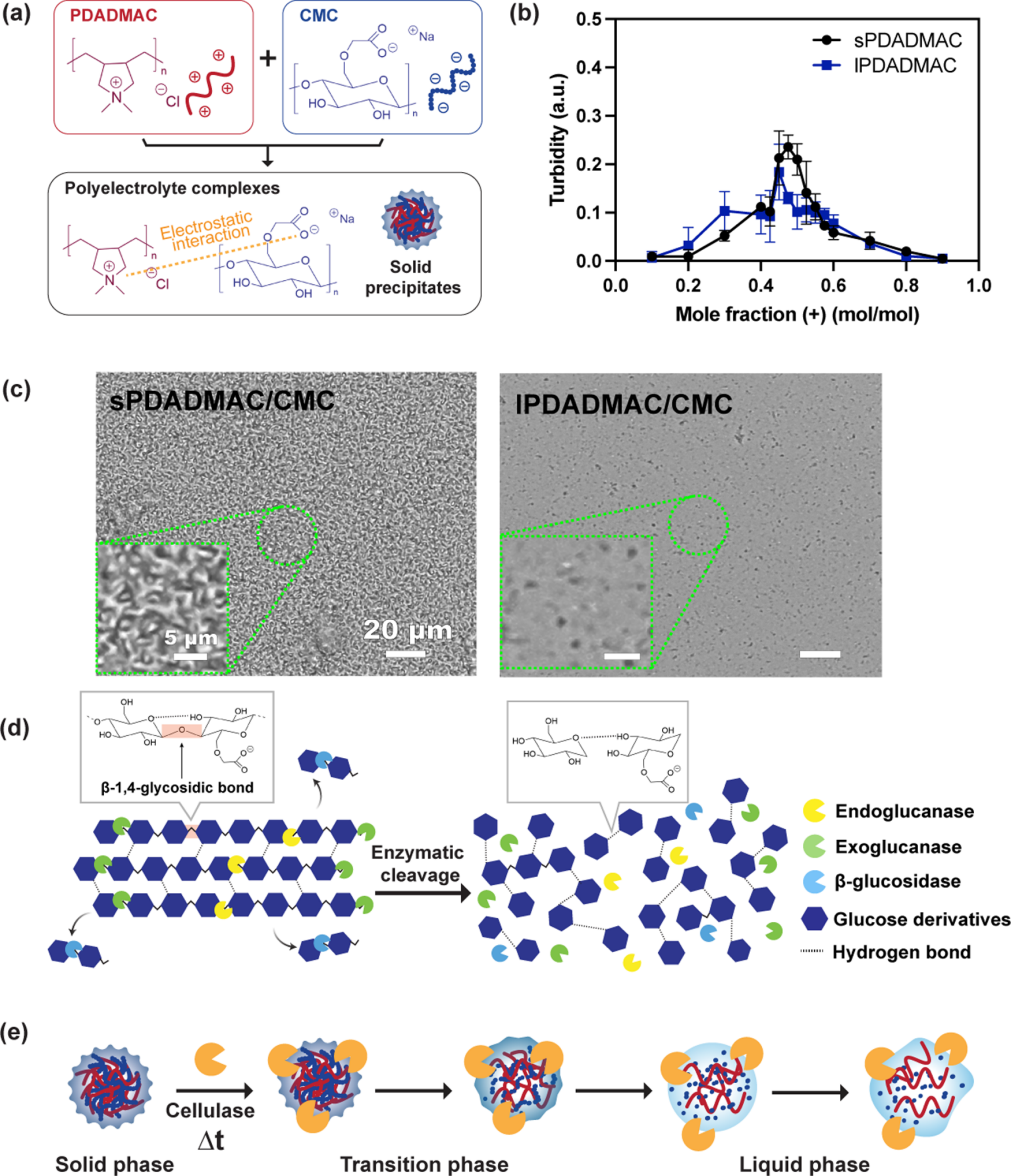
(a) Schematic image of the polyelectrolyte complexation driven by electrostatic interactions between PDADMAC and CMC at pH 7.0. (b) Plot of turbidity of short and long PDADMAC/CMC complexes as a function of positive mole fraction/total mole fraction at 3 mM total polymer concentration. Error bars represent the standard deviation of triplicate runs. (c) Brightfield micrographs of sPDADMAC/CMC (left) and lPDADMAC/CMC (right) at 47.5/52.5 mol % and 3 mM total polymer. Insets show magnified views. Scale bars indicate 20 μm (main) and 5 μm (inset). (d) A schematic illustration of the cellulase action mechanism and (e) a schematic drawing showing enzyme-induced transitions in PDADMAC/CMC complexes.

### 2.4 Turbidity Measurement

Turbidity measurements were used to characterize both the formation and the enzyme-induced transitions of the PDADMAC/CMC complexes. Measurements were performed using a plate reader equipped with a UV spectrophotometer (Agilent Technologies, Inc., Cytation 5, CA, USA) at a wavelength of 562 nm, where none of the polymers absorb light. Turbidity results from light scattering by polyelectrolyte complexes, and is described by ln­(*I*/*I*_0_), where *I*_0_ represents the incident light intensity, and *I* denotes the intensity of the light that passes through the sample volume measured in absorption units (a.u.). The PDADMAC/CMC mixtures were placed into each well of a 384- or 96-well plate. Triplicate experiments were conducted, and the average turbidity signal was calculated.

### 2.5 Brightfield Microscopy

An optical microscope (ECHO, Rebel, CA, USA) was used to observe the morphologies of the PDADMAC/CMC complexes. After the enzyme addition, time-lapse images were acquired to monitor morphological changes.

### 2.6 Zeta Potential Measurement

Zeta potential measurements were used to assess how cellulase concentration alters the surface charge of PDADMAC-based systems including PDADMAC/cellulase and PDADMAC/CMC mixtures. Measurements were performed using a Zetasizer Nano ZS instrument (Malvern Panalytical, Malvern, UK). Samples were prepared in 10 mM HEPES buffer at pH 7.0, and cellulase was added to 0, 0.04, 0.2, and 0.8 mM. After adding cellulase, we gently mixed the mixtures prior to loading. Four sample types were analyzed including sPDADMAC/cellulase (SP), sPDADMAC/CMC (SPC), lPDADMAC/cellulase (LP), and lPDADMAC/CMC (LPC). Samples were loaded into folded capillary cells (DTS1070, Malvern Panalytical) and equilibrated for 3 min at 25 °C before measurement. Triplicate experiments were conducted, and each measurement consisted of three runs with at least 12 data points recorded per run and up to 100 data points recorded per run as needed.

### 2.7 Rheological Measurements

Rheology measurements were performed using a Discovery Hybrid Rheometer 20 instrument (HR-20, TA Instruments, DE, USA) equipped with a Peltier temperature control system. The top geometry was an 8 mm parallel plate, and the bottom geometry was a Peltier plate. sPDADMAC and lPDADMAC systems were prepared following the same protocol described in Table S2, in batches totaling 300 mL, using 500 mL centrifuge bottles. The mixtures were centrifuged at 8,000 g for 20 min and aged for 24 h. Our visual observations of the sample follow the same trends as those of the lPDADMAC system (Figure S3a). After removal of the supernatant, the dense phase that settled at the bottom was transferred to 1.5 mL Eppendorf tubes and centrifuged again for 5 min. The supernatant was removed, and this step was repeated once more for 5 min to further remove the residual supernatant. The resulting dense phases were loaded onto the bottom geometry for measurements (Figure S3b). Frequency-sweep measurements were performed at a strain amplitude of 1%, which was confirmed to be within the linear viscoelastic region by amplitude-sweep tests using samples prepared without cellulase. Samples containing 0, 0.02, 0.08, and 0.8 mM cellulase were measured at 2, 24, and 72 h after enzyme addition to monitor enzyme- and time-dependent changes in viscoelastic behavior.

### 2.8 Statistical Analysis

The error bars in all graphs represent the standard deviation determined from the variation in repeated technical and biological experiments and corresponding calculations. A total of three biological and three technical replicates for each system, with three reads per replicate, results in *N* = 27 for turbidity measurements. Zeta potential measurements had three biological and three technical replicates, yielding 12–100 data points per sample (total *N* = 108–900, depending on signal stability). Rheological measurements were performed in three biological replicates.

## 3 Results and Discussion

### 3.1 Polyelectrolyte Complexation

We formed PDADMAC/CMC complexes at pH 7.0 using CMC with a degree of substitution (DS) of 0.6 and two PDADMACs differing in chain length: short PDADMAC (sPDADMAC) and long PDADMAC (lPDADMAC) (Figure 1a). PDADMAC is a strong, positively charged polyelectrolyte, while CMC is a weak, negatively charged polyelectrolyte derived from cellulose. Because PDADMAC contains a quaternary ammonium group per monomer, it carries a charge over a wide pH range and is largely insensitive to pH changes. In contrast, CMC contains carboxymethyl groups whose ionization is pH-dependent and is therefore commonly considered a weak polyelectrolyte with an apparent p*K*_a_ of ∼ 4–4.6, depending on DS and solution conditions.^32,33^ CMC is a linear polysaccharide composed of anhydro-glucose linked by β-1,4-glycosidic bonds and has a varying DS, where the DS represents the average number of carboxymethyl groups substituted per monomer unit.^34,35^ While the theoretical maximum DS of CMC is 3, indicating complete substitution of all hydroxy groups in each monomer,^36^ commercially available CMC typically has a DS between 0.4 and 1.5, with the remaining unsubstituted positions occupied by hydrogen.^34,36^ The DS influences the solubility, viscosity, and salt resistance of CMC.^37^

To define the composition that maximizes complex formation, we prepared mixtures across the mole fraction of PDADMAC (*x*_PDADMAC_) at a fixed total polymer concentration of 3 mM and used turbidity measurements to determine the mole fraction at which maximum complexation occurs. After sample preparation, the plate was sealed and allowed to settle for 24 h at room temperature to establish a stable baseline in the absence of an enzyme. The resulting turbidity graph revealed distinct maxima for the two PDADMAC chain lengths. The sPDADMAC system peaked at *x*_PDADMAC_ = 0.475, whereas the lPDADMAC system peaked at *x*_PDADMAC_ = 0.45 (Figure 1b). Thus, in both cases, optimal complex formation occurred at slightly PDADMAC-deficient compositions. Both systems appeared as solid precipitates rather than liquid coacervates, as also evident in the micrographs taken at 47.5/52.5 mol % PDADMAC/CMC (Figure 1c).

The sPDADMAC complexes appear as densely packed precipitate. In contrast, the lPDADMAC sample shows fewer aggregates, consistent with its lower turbidity intensity and with the 47.5/52.5 mol % composition being slightly offset from its turbidity optimum at *x*_PDADMAC_ = 0.45. To compare chain length effects under the same starting conditions, we fixed the starting composition at the sPDADMAC turbidity maximum for both chain lengths. We used *x*_PDADMAC_ = 0.475 at 3 mM total polymer and pH 7.0 for both systems, which produced comparable initial solid precipitates. This approach isolates the enzyme response to chain length rather than shifts in optimum stoichiometry.

### 3.2 Cellulase-Induced Phase Transition

#### 3.2.1 Turbidity and Brightfield Microscopy

We next examined the structural transition of PDADMAC/CMC complexes at pH 7.0 using two PDADMAC chain lengths at a total polymer concentration of 3 mM. The sPDADMAC had an average molecular weight of ∼8.5 kDa, corresponding to an estimated chain length of *N* ∼ 53, whereas the lPDADMAC had an average molecular weight of <100 kDa, corresponding to *N* < 618. For comparison, we fixed the starting composition at *x*_PDADMAC_ = 0.475, which corresponds to the turbidity maximum for the sPDADMAC system (Figure 1b).

After baseline turbidity readings, we investigated how the complexes' light scattering would change after cellulase addition. Figure 1d summarizes how cellulase interacts with the CMC backbone. Cellulase binds to CMC at the catalytic sites and cleaves β-1,4-glycosidic bonds.^38^ Cellulase is typically composed of three classes of enzymes, namely endoglucanase, exoglucanase, and β-glucosidase.^39^ While these enzymes serve the same overall function of CMC degradation, they act at different sites within the polymer chain. Endoglucanases and exoglucanases initiate the degradation of the CMC, producing shorter polymers that can form oligomers of a few glucose monomers. Specifically, endoglucanase randomly cleaves the β-1,4-glycosidic bond between glucose monomers in the CMC chain, releasing oligosaccharides of varying lengths.^38,40^ Exoglucanase primarily acts at the ends of the CMC chains, producing cellobiose.^38^ β-glucosidase then finalizes the hydrolysis by converting cellobiose into d-glucose.^41^ This sequence shortens the CMC and weakens ionic interactions that stabilize the solid complex. Figure 1e outlines the polyelectrolyte complex model in which chain scission reduces connectivity and the complex softens as chains are cleaved and become a liquid-like material.

We tracked this transition with 72 h of turbidity kinetics on preformed complexes. In the absence of cellulase, the turbidity remained constant over time, indicating a stable solid precipitate. In the sPDADMAC system, cellulase addition decreased monotonically (Figure 2a), although at 0.8 mM cellulase, it exhibited a brief early time plateau before decreasing. In the lPDADMAC system, turbidity decreased gradually at concentrations lower than 0.4 mM (Figure 2b). In contrast, at 0.4 and 0.8 mM, turbidity increased at early times (peaking around ∼ 1 h) before decreasing at later times. Compared to no enzyme, the time-dependent decrease in turbidity after cellulase addition likely reflects CMC chain scission and the resulting weakening of ionic interactions with the nondegradable PDADMAC. However, the transient rise/plateau could not be further interpreted from turbidity measurements alone. Thus, we next used microscopy to directly visualize the evolution of the complex’s morphology over time after enzyme addition.

**Figure 2:**
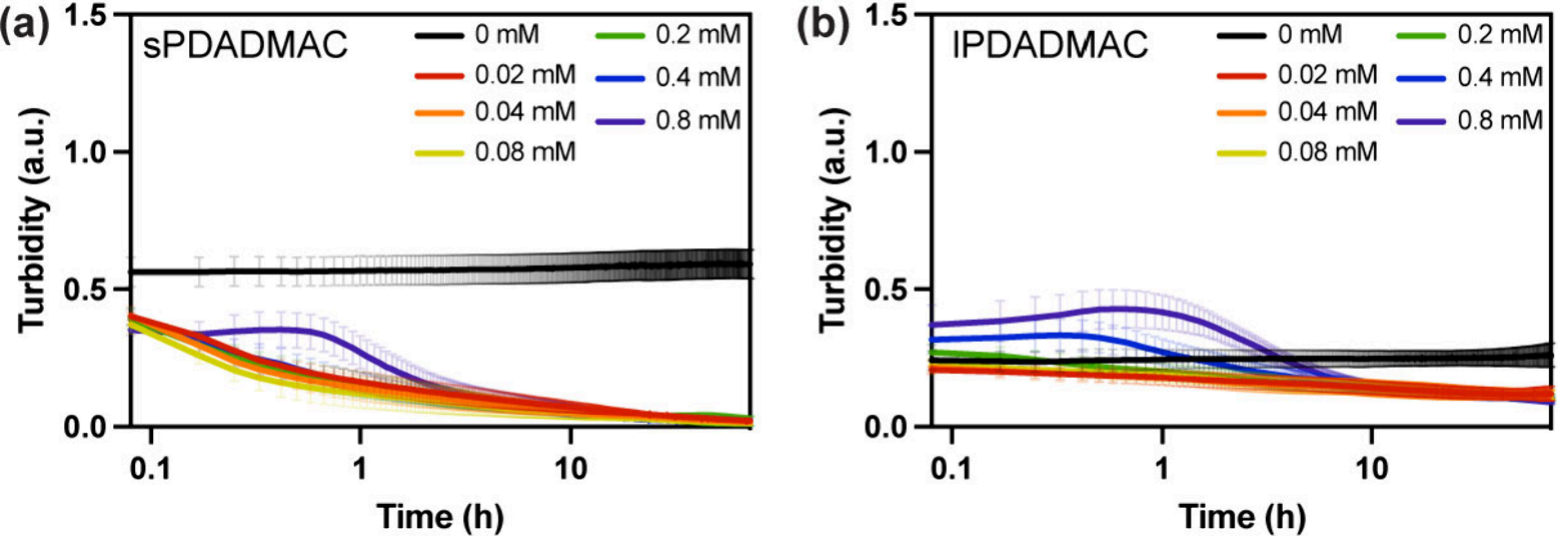
Turbidity as a function of post-enzyme addition time (log scale) for (a) sPDADMAC/CMC and (b) lPDADMAC/CMC complexes in the presence of cellulase at 0, 0.02, 0.04, 0.08, 0.2, 0.4, and 0.8 mM.

Brightfield imaging captured the enzyme-induced morphological evolution for both chain lengths (Figure 3). We first monitored the sPDADMAC/CMC system at a 47.5/52.5 mol ratio with 0.02 mM cellulase by 72 h (Figure S1). Before enzyme addition, densely packed precipitates were present. After addition, the precipitates progressively softened and relaxed over time. Notably, the material did not fully dissolve; instead, it appeared to wet and spread along the bottom of the well. We attribute this wetting-like behavior to cellulase-driven cleavage of CMC, which reduces electrostatic interactions between PDADMAC and CMC, thereby allowing the complexes to relax rather than fully disperse. This enzymatic cleavage is also expected to increase water uptake because loosening ion pairing facilitates water to penetrate the complexes more readily. Increased hydration can further facilitate relaxation of ion pairing.^42^ Consistent with this idea, several studies have shown that water can plasticize PECs by increasing the free volume and enabling chain motion.^42–45^ Thus, we expect that water contributes not only to glycosidic bond cleavage during cellulase-catalyzed hydrolysis but also as a plasticizer that promotes the subsequent phase transition.^46,47^ As the enzyme concentration increased, we observed the samples wetting the surface more quickly, indicating a more liquid-like state. At 0.8 mM cellulase, sPDADMAC precipitates were rounded and broke up within 30 min (Figure 3a,b). They then produced some small complexes that grew and intermittently coalesced after 72 h. Meanwhile, we observed fewer complexes between lPDADMAC and CMC, but a clear transition from precipitates to liquid droplets at all enzyme concentrations (exemplified in Figure 3c). In addition, lPDADMAC showed a rapid post-30 min increase in droplet number and a polydisperse population that persisted through 72 h (Figure 3c). This behavior may arise because longer PDADMAC carries a larger number of charges per chain, providing more interaction sites for cellulase and thereby promoting stronger partitioning of cellulase into the polymer-rich phase and accelerated droplet formation.^48^

**Figure 3:**
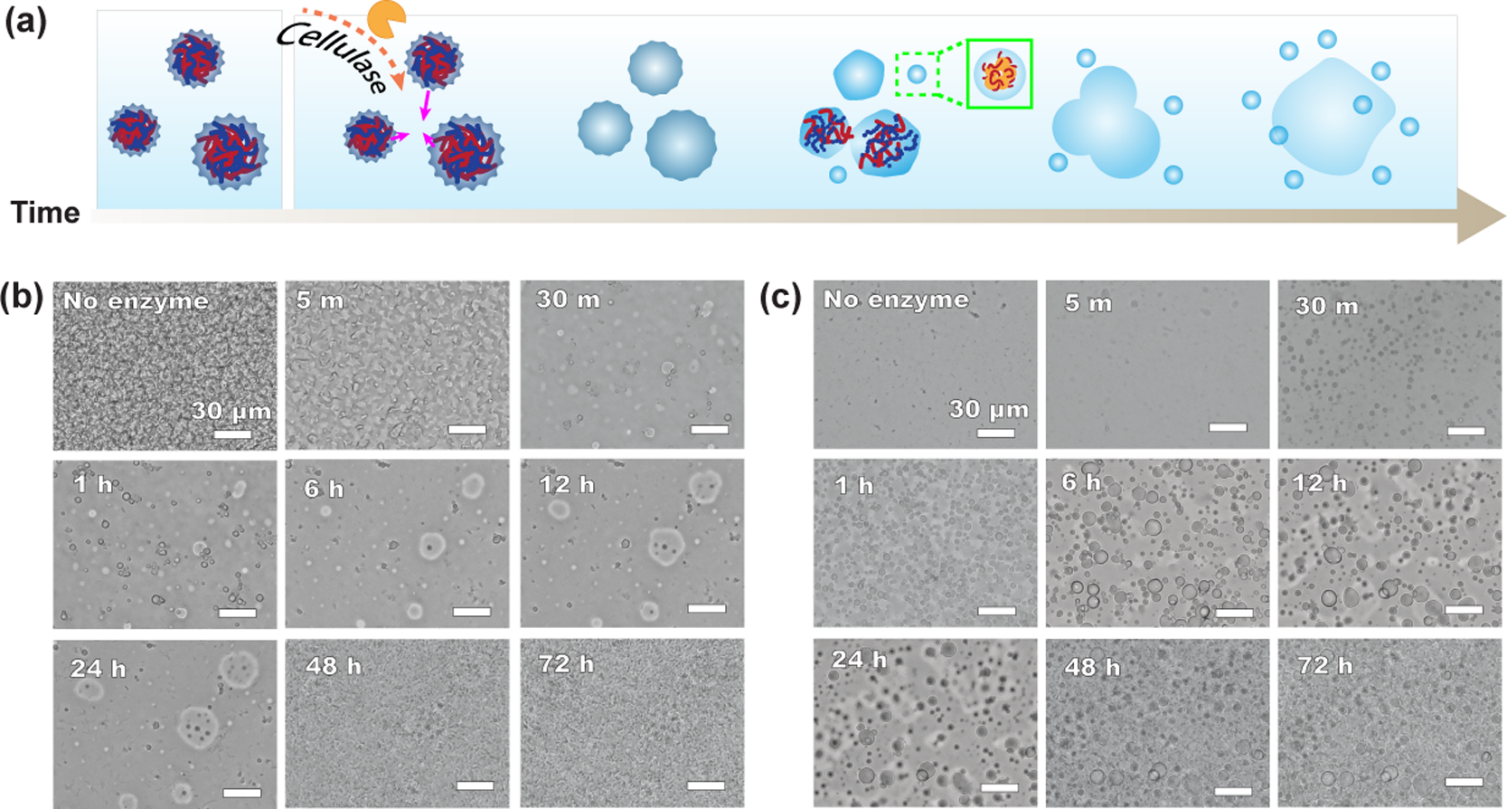
(a) Schematic illustration of the morphological evolution of PDADMAC/CMC complexes over time after cellulase addition. Enzymatic cleavage of the CMC chain relaxes solid precipitates, while secondary PDADMAC-cellulase coacervates (inside the green box) form and grow with time. Brightfield micrographs of (b) sPDADMAC/CMC and (c) lPDADMAC/CMC complexes at 47.5/52.5 mol % and 3 mM total polymer, recorded from before enzyme addition to 72 h at 0.8 mM cellulase. The scale bar is 30 μm.

Based on bright-field results, we found that small coacervates appear early at higher enzyme concentration. We hypothesize that these small coacervates cause a transient plateau or a rise in turbidity at high enzyme concentrations, especially in the lPDADMAC system. To test this, we examined PDADMAC/cellulase mixtures and compared them with those of the PDADMAC/CMC system. In the sPDADMAC mixtures, turbidity rose over the first 2 h at 0.4 to 0.8 mM enzyme and then relaxed (Figure 4a). The lPDADMAC mixtures behaved similarly but with more pronounced peaks and a slower decay (Figure 4b). Additionally, at 2 h, we observed that PDADMAC/cellulase complexes increased in number and size with increasing enzyme concentration and appeared denser for longer PDADMAC (Figure 4c). These observations are consistent with a transient rise in light scattering in the turbidity driven by the rapid formation and coarsening of small coacervates. We confirmed that these droplets arise from electrostatic interaction by ζ-potential shifts (Figure 4d). At pH 7.0, PDADMAC/CMC complexes are net positive, and cellulase is net negative (overall charge of −52.7 or −9.8, see SI, section 4). With increasing enzyme concentration, ζ-potentials shift toward more negative values for both the PDADMAC/cellulase mixtures (e.g., sPDADMAC/cellulase and lPDADMAC/cellulase) and the PDADMAC/CMC complexes (sPDADMAC/CMC and lPDADMAC/CMC). Thus, these results indicate electrostatic binding of cellulase to PDADMAC and a concurrent reduction in the free polycation available for CMC bridging, explaining the transient increase in scattering followed by a later decrease as chain scission proceeds.

**Figure 4:**
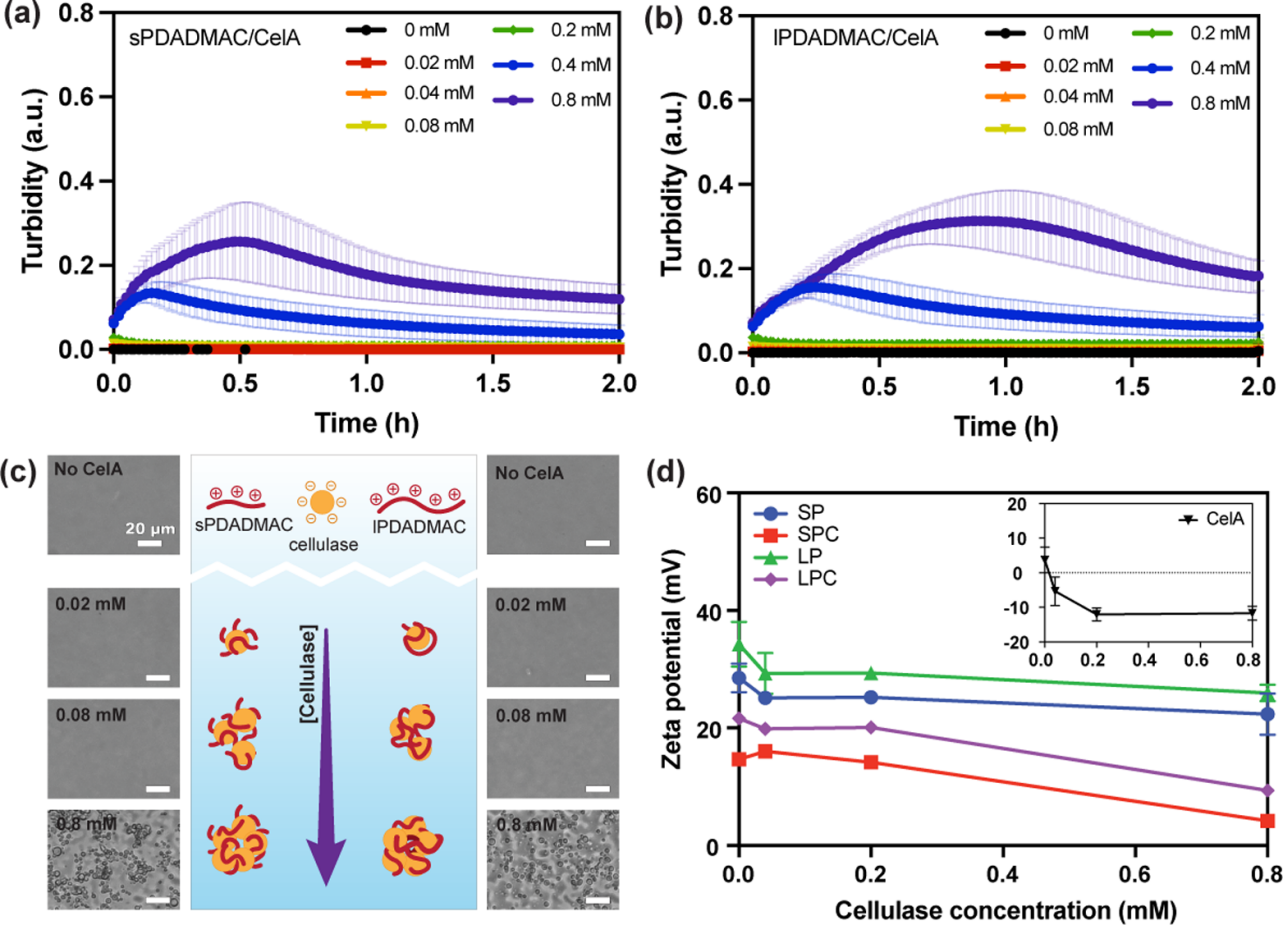
Turbidity-time profiles for complexes formed between cellulase and sPDADMAC (a) and lPDADMAC (b) over 2 h at cellulase concentrations of 0, 0.02, 0.04, 0.08, 0.2, 0.4, and 0.8 mM. (c) Brightfield microscopy images of sPDADMAC/cellulase (left) and lPDADMAC/cellulase (right) after 2 h at the indicated concentrations, with a schematic illustrating a distinct complex structure formed with cellulase by chain length. Scale bars represent 20 μm. (d) ζ-potential as a function of cellulase concentration for sPDADMAC/cellulase (SP), sPDADMAC/CMC (SPC), lPDADMAC/cellulase (LP), and lPDADMAC/CMC (LPC) complexes. The inset shows the ζ-potential of cellulase solutions alone (CelA). All samples were prepared at 3 mM total polymer and 47.5/52.5 mol % PDADMAC/CMC at pH 7.0. Error bars represent the standard deviation of triplicate runs.

To confirm whether PDADMAC/cellulase coacervates persist beyond 72 h, we extended imaging of the sPDADMAC/CMC system to 3 weeks across enzyme concentrations (Figure S2). At 0.4 and 0.8 mM cellulase, PDADMAC/cellulase droplets were still visible at days 3, 7, 14, and 21. Compared with earlier times, the images suggest fewer visible droplets, but apparent changes in droplet counts and sizes could not be confirmed from these micrographs.

#### 3.2.2 Rheology of the Enzyme-Induced Transitions

Although we visually observed the formation of liquid droplets, we next quantified the linear viscoelastic response by rheology to connect the optical and morphological changes to the material behavior. Frequency sweeps probed samples at *x*_PDADMAC_ = 0.475 with 3 mM total polymer in a 10 mM HEPES, pH 7.0 solution at 2, 24, and 72 h after cellulase addition at 0, 0.02, 0.08, and 0.8 mM cellulase concentration. This data set captures enzyme- and time-dependent evolution within each chain-length system while directly comparing short- and long-chain PDADMAC at matched enzyme concentrations and postenzyme times.

In the absence of cellulase, both systems were solid-like as *G*′ exceeded *G*″ across the frequency range at 2, 24, and 72 h (Figures 5a and S4a). The moduli also showed only modest time dependence, indicating that the PDADMAC/CMC structure remained mechanically stable on this time scale, in the absence of enzymatic cleavage. Upon cellulase addition, both systems softened with the enzyme concentration and time. This trend suggests that cellulase-driven scission of the CMC reduces the effective chain length of the polyanion and alters interpolymer interactions (e.g., electrostatic interactions and hydrogen bonding) within the PEC structure. For sPDADMAC/CMC, *G*′ and *G*″ decreased substantially by 72 h, and the reduction was most noticeable at higher cellulase concentrations (Figure 5b–d). Notably, at matched enzyme concentrations, *G*′ already decreased in magnitude from 2 to 24 h, indicating time-dependent softening before the larger reductions observed at 72 h (Figure S5a). The lPDADMAC/CMC system exhibited the same overall softening trend (Figure S4b–d), but the 2–24 h decrease was more gradual (Figure S5b). Across matched enzyme concentrations and post-enzyme times, lPDADMAC/CMC generally maintained a higher *G*′ than sPDADMAC/CMC once the enzyme was present (Figure S6). At 72 h, the *G*′–*G*″ crossover point (highlighted by red circles) occurred at a lower frequency in the lPDADMAC system than in the sPDADMAC system (Figures 5c,d and S4).

**Figure 5:**
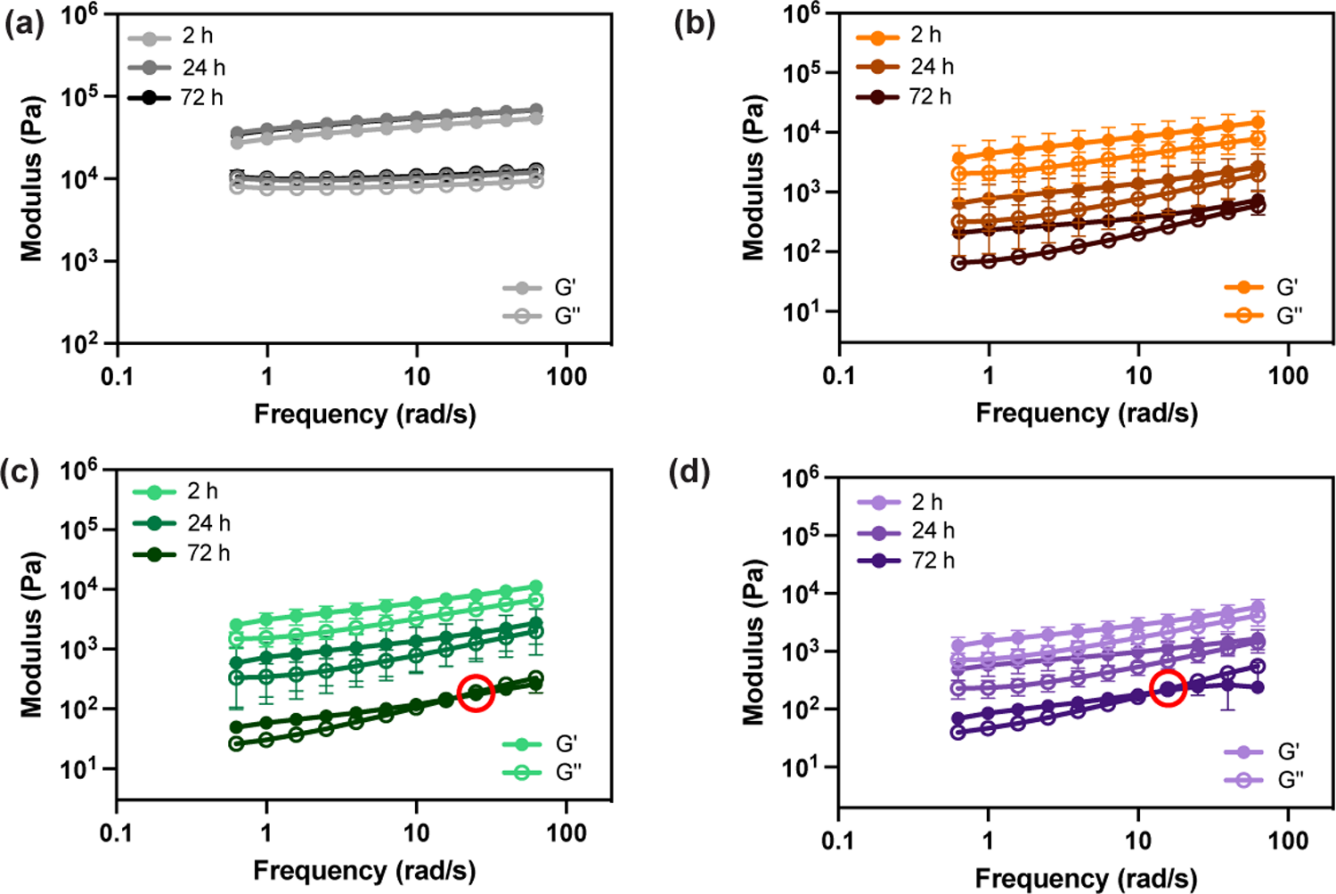
Frequency sweeps of sPDADMAC/CMC at a 47.5/52.5 mol ratio and 3 mM total polymer. Storage modulus (closed symbols) and loss modulus (open symbols) as a function of angular frequency for samples containing (a) 0 mM, (b) 0.02 mM, (c) 0.08 mM, and (d) 0.8 mM cellulase, measured at 2, 24, and 72 h after enzyme addition. Red circles indicate *G*′–*G*″ crossover. All measurements were conducted at pH 7.0. Error bars represent the standard deviation of triplicate runs.

The results above suggest that the long-chain system retains a longer characteristic relaxation time at late stages of cellulase-driven structural evolution. Previous studies reported that relaxation time and viscosity are associated with chain length, noting that a *G*′–*G*″ crossover at higher frequency corresponds to shorter relaxation times and faster, largely unentangled chain dynamics.^13,24,25,49^ In contrast, a crossover at a lower frequency indicates longer relaxation, which can arise as longer chains form intermolecular entanglements that restrict chain motion. In our PECs, however, chain-length mismatch is also relevant because a fixed long CMC chain (*N* = 1050) is paired with shorter PDADMAC chains (*N* ∼ 53 or *N* < 618). Spruijt et al. suggested that the polycation chain length can strongly influence complex dynamics, while the polyanion may primarily act as transient, sticky cross-links.^25^ Liu et al. further reported that in asymmetric length complexes, viscoelasticity can be biased toward the short chain, attributed to its larger translational entropy per charge.^24^ These findings suggest that a shift in crossover frequency in our system could reflect PDADMAC chain length dynamics. An additional factor that may contribute to these trends is the formation of PDADMAC/cellulase coacervates, which appears to be more pronounced in the lPDADMAC system. As cellulase partitions into a PDADMAC-rich phase, the effective enzyme availability within the PEC could be reduced, potentially slowing CMC scission and delaying mechanical relaxation. In addition, because this newly formed coacervate is expected to be more viscous than the surrounding aqueous phase, its increasing volume fraction may also contribute directly to the bulk viscoelastic response.

Lastly, we compared the complex viscosities of both systems using the same frequency-sweep data sets (Figure S7a–d). Viscosities decreased with cellulase concentration and postenzyme time for both systems, which align with decreasing moduli trends. As observed, lPDADMAC/CMC generally retains a higher viscosity than that of sPDADMAC/CMC, highlighting that the long-chain system maintains greater resistance to flow even as the complexes soften.

## 4 Conclusion

In this study, we showed that initially solid PDADMAC/CMC complexes can be driven toward more liquid-like materials via enzymatic cleavage of CMC by cellulase. We first formed precipitates at a fixed total polymer concentration and mole fraction. Upon cellulase addition, cleavage of the β-1,4-glycosidic bonds shortened CMC, weakened electrostatic interactions, and enabled the complexes to relax from rigid solids to liquid-like materials. Time-resolved turbidity and bright-field microscopy captured this evolution. In the absence of the enzyme, the complexes remained stable and were highly turbid. With increasing enzyme dose, they became progressively wetted and rounded, approaching a liquid-like morphology.

Comparing short and long PDADMAC highlighted how the polycation chain length regulates this enzymatic response. For sPDADMAC/CMC, the overall turbidity decreased after enzyme addition and reached a plateau at a relatively low cellulase concentration. In contrast, lPDADMAC/CMC showed a transient increase in turbidity at mid-to-high enzyme concentrations and reached the plateau more slowly. Microscopy and turbidity measurements suggested that this nonmonotonic behavior arises from a competing process in which PDADMAC/cellulase coacervates form, which transiently increase light scattering and may redistribute polycation away from the original complexes. Consistent with this interpretation, the zeta potential measurement of PDADMAC-cellulase mixtures in the absence of CMC confirmed the interaction between PDADMAC and cellulase, supporting a binary electrostatic association that can reduce the effective polycation available for CMC bridging during the early stages of the transition.

Rheology measurements provided a quantitative measure of the mechanical response associated with these transitions. In the absence of cellulase, both chain-length systems behaved like solids, with *G*′ exceeding *G*″ across the probed frequencies and showing minimal time dependence. After enzyme addition, *G*′ and *G*″ decreased with increasing cellulase concentration and post-enzyme time, indicating a move toward more liquid-like behavior. At matched enzyme concentrations and times, lPDADMAC complexes retained higher *G*′ and complex viscosity than sPDADMAC. They exhibited *G*′–*G*″ crossovers at lower frequency, indicating that longer chains maintain slower relaxation as the complexes soften.

Collectively, our findings provide a coherent picture in which enzymatic cleavage of the biopolymer relaxes solid PECs and tunes their mechanical response. The polycation chain length and enzyme concentration together govern whether the system undergoes a solid-to-liquid transition of the complexes or instead forms secondary enzyme-polycation coacervates. More broadly, these findings show that enzymes are not only catalytic triggers but also charged macromolecular components that reshape the PEC phase behavior. Thus, localized enzymatic activity provides a microscale handle to program the remodeling of solid PECs beyond bulk stimuli.

## Supplementary Material



## Notes

Experimental procedures of polyelectrolyte complexation and cellulase-induced phase transition; Bright-field microscope images of sPDADMAC/CMC complexes upon cellulase addition; Enzyme net charge calculation by MATLAB; Sample preparation for rheological measurements; Frequency sweep and complex viscosity data of short and long PDADMAC/CMC (PDF)

## Abbreviations

PEC(s): polyelectrolyte complex­(es)
CMC: carboxymethyl cellulose
PDADMAC: poly­(diallyldimethylammonium chloride)
sPDADMAC: short poly­(diallyldimethylammonium chloride)
lPDADMAC: long poly­(diallyldimethylammonium chloride)
DS: the degree of substitution
*x*_PDADMAC_: mole fraction of PDADMAC
