# Correction to “Enzyme-Induced Transition of the Morphology of Polyelectrolyte Complexes”

**DOI:** 10.1021/acs.biomac.6c00872

**Published:** 2026-07-11

**Authors:** Chaeyoung Lim, Whitney C. Blocher McTigue

The structure of the carboxymethyl cellulose (CMC) in Figure 1­(d) was incorrectly drawn. Please find the corrected Figure 1(d) graphic below.

**Figure 1:**
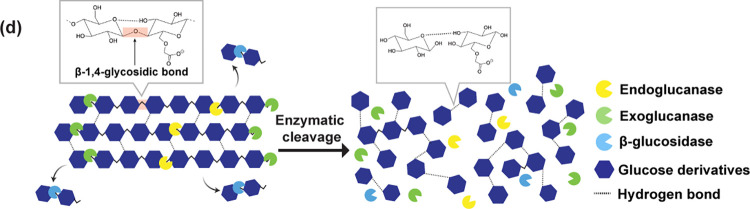
(d) A schematic illustration of the cellulase action mechanism.

