# Supplementary material for: Enzyme-Induced Transition of the Morphology of Polyelectrolyte Complexes
Source: Biomacromolecules. 2026 Feb 13;27(3):2343–51. doi: 10.1021/acs.biomac.6c00029 (PMC12977053; doi:10.1021/acs.biomac.6c00029)
Supplement: Supplementary file 1 [file bm6c00029_si_001.pdf]

Supporting Information

Enzyme-Induced Transition of the Morphology of Polyelectrolyte Complexes

Chaeyoung Lim and Whitney C. Blocher McTigue*

Department of Chemical and Biomolecular Engineering, Lehigh University, PA 18015, United States

*Corresponding author: whb322@lehigh.edu

1. Polyelectrolyte Complexation

To examine the optimal mole fraction where PDADMAC/CMC complexation occurs at maximum, the mole fraction of PDADMAC (x_{PDADMAC}) was systematically varied from 0.1 to 0.9 (eq. 1), and for each mole fraction, PDADMAC/CMC complexes were prepared at 3 mM total polymer concentration, on a monomer basis.

$$x_{\mathrm{PDADMAC}} = \frac{[\mathrm{PDADMAC}]}{[\mathrm{PDADMAC}] + [\mathrm{CMC}]} \quad (1)$$

Note that PDADMAC has one charge per monomer unit, whereas each anhydro-glucose unit of CMC contains three hydroxyl groups, of which approximately 0.6 are substituted with a carboxymethyl group.

Table S1. Formulations used to prepare short and long PDADMAC/CMC mixtures at 3 mM total polymer concentration and various x_{PDADMAC} in 10 mM HEPES buffer at pH 7.0. For each formulation, a total volume of 120 μL was prepared and aliquoted into three Eppendorf tubes (35 μL per tube) for biological triplicate measurements. A background (blank) sample was prepared using HEPES buffer and water only; its signal was used for background correction.

Sample #	Mole fraction of PDADMAC (+) (mol/mol)	Volume of 10 mM PDADMAC stock (+) (μL)	Volume of 10 mM CMC stock (-) (μL)	Volume of 10 mM HEPES buffer stock (μL)	Volume of DI Water (μL)
1	0.100	3.6	32.4	2.4	81.6
2	0.200	7.2	28.8		
3	0.300	10.8	25.2		
4	0.400	14.4	21.6		
5	0.425	15.3	20.7		
6	0.450	16.2	19.8		
7	0.475	17.1	18.9		
8	0.500	18.0	18.0		
9	0.525	18.9	17.1		
10	0.550	19.8	16.2		
11	0.575	20.7	15.3		
12	0.600	21.6	14.4		
13	0.700	25.2	10.8		
14	0.800	28.8	7.2		
15	0.900	32.4	3.6		

2. Cellulase-Induced Phase Transition

This study examined the effects of enzyme concentration on the solid-to-liquid phase transition of PDADMAC/CMC complexes. Samples were prepared at x_{PDADMAC} = 0.475 and total polymer concentration was 3 mM, in 10 mM HEPES buffer at pH 7.0. Table S2 summarizes the composition in which various concentrations of cellulase (0, 0.02, 0.04, 0.08, 0.2, 0.4, and 0.8 mM) were added to pre-formed PDADMAC/CMC complexes prepared at 3 mM total polymer concentration. After preparation, the samples were transferred into a 96-well plate for turbidity measurements over 72 hours. The samples with varying enzyme concentrations were used to investigate how cellulase affects the solid-to-liquid phase transition of PDADMAC/CMC complexes at 3 mM total polymer concentration.

Table S2. Formulations used to prepare short and long PDADMAC/CMC mixtures at 3 mM with x_{PDADMAC} = 0.475 in 10 mM HEPES buffer at pH 7.0. For each formulation, a total volume of 650 μL was prepared without cellulase and aliquoted into three biological replicates. Each replicate was transferred to a 96-well plate at pre-cellulase volume (200 μL minus the cellulase addition volume) and aged overnight to allow complexes to settle. Cellulase was then added to each well immediately prior to measurement to reach a final volume of 200 μL.

Sample #	Volume of 10 mM PDADMAC stock (+) (μL)	Volume of 10 mM CMC stock (-) (μL)	Volume of 10 mM cellulase added (μL)	Volume of 10 mM HEPES buffer stock (μL)	Volume of DI water (μL)
1	92.6	102.4	0	13	442.0
2			1.0		441.0
3			2.6		439.4
4			5.1		436.9
5			10.2		431.8
6			25.6		416.4
7			51.2		390.8

3. Bright-Field Microscopy

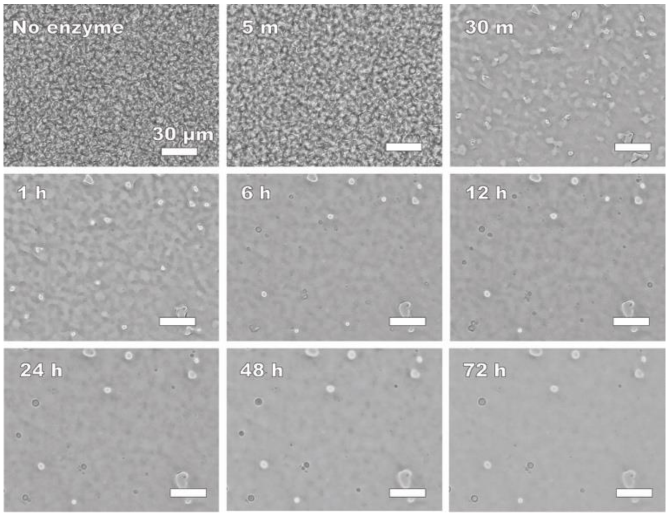

Figure S1. Bright-field micrographs of sPDADMAC/CMC complexes at 47.5/52.5 mol% and 3 mM total polymer, recorded from before enzyme addition to 72 h at 0.02 mM cellulase. The scale bar represents 30 μm.

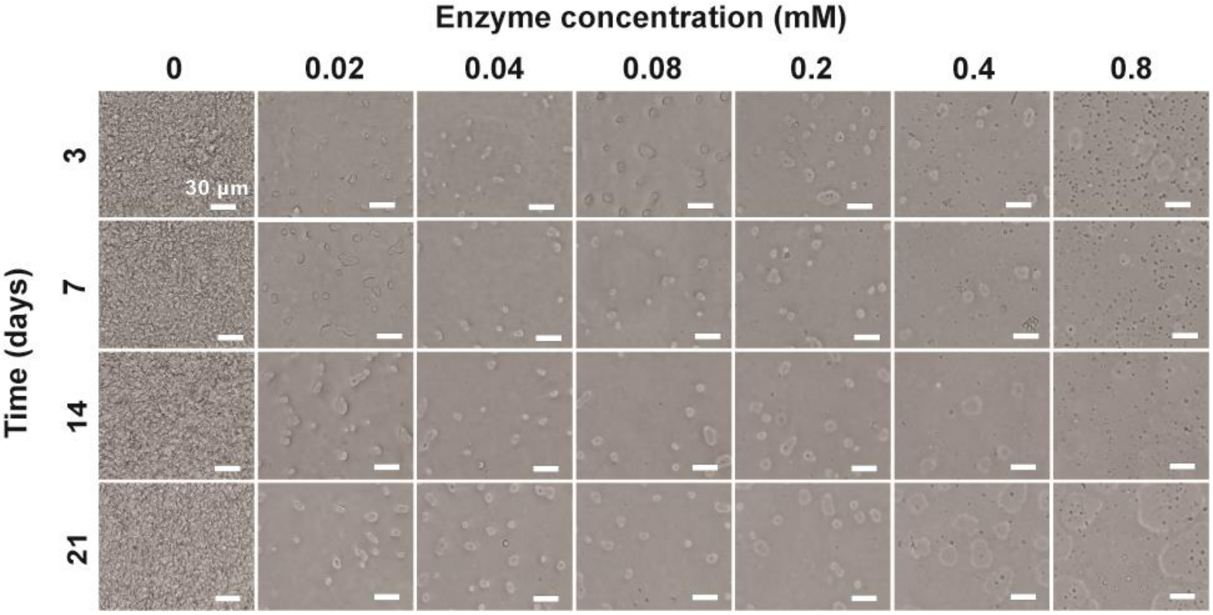

Figure S2. Bright-field micrographs of sPDADMAC/CMC complexes at 47.5/52.5 mol% and 3 mM total polymer over a concentration series of 0, 0.02, 0.04, 0.08, 0.2, 0.4, and 0.8 mM, captured

up to 3 weeks. The scale bars represent 30 μm. Note that the images were not taken at identical sample coordinates.

4. Enzyme Net Charge Calculation

We used a MATLAB script (below) and the Henderson-Hasselbalch equation to calculate the net charge of our cellulase. We used 5E09 (-52.7 charge) and 5061 (-9.8 charge) from the Protein Data Bank[1] as our cellulase sequences.

5. Rheological Measurement

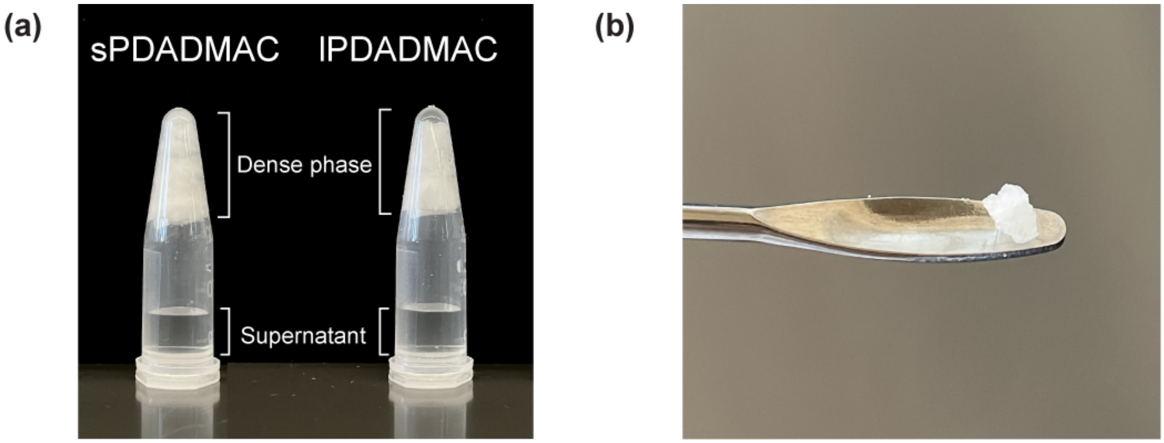

Figure S3. Visual observation of sPDADMAC and lPDADMAC complexes at 47.5/52.5 mol% and 3 mM total polymer. **(a)** A photograph of phase-separated samples prepared by mixing sPDADMAC and lPDADMAC with CMC at pH 7.0, showing a distinct dense phase and a supernatant. **(b)** A photograph of the dense phase collected using a spatula from sPDADMAC/CMC complexes. The material is slightly elastic and resembles a soft white solid.

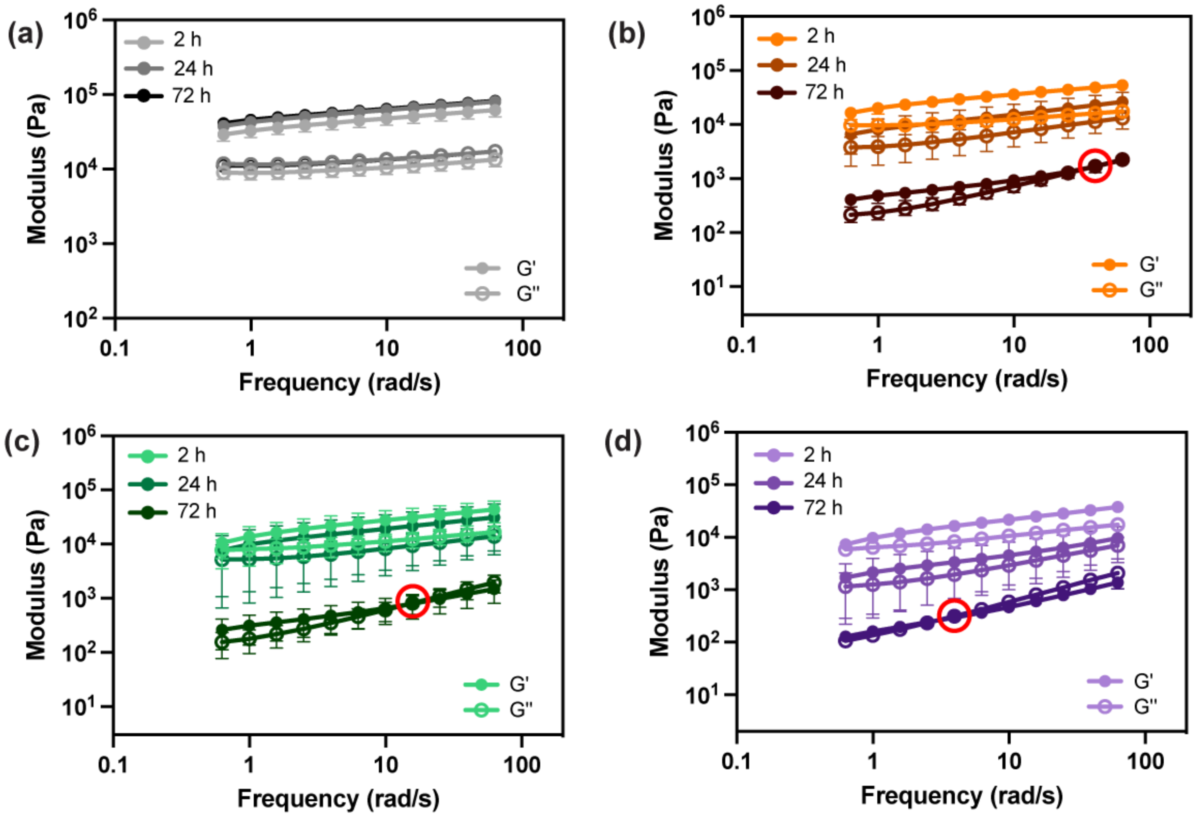

Figure S4. Frequency sweeps of the lPDADMAC/CMC. Storage modulus (closed symbols) and loss modulus (open symbols) as a function of angular frequency for samples containing **(a)** 0 mM, **(b)** 0.02 mM, **(c)** 0.08 mM, and **(d)** 0.8 mM cellulase, measured at 2, 24, and 72 h after enzyme addition. Red circles indicate $G' - G''$ crossover where tan $\delta = 1$. Error bars represent the standard deviation of the triplicate runs.

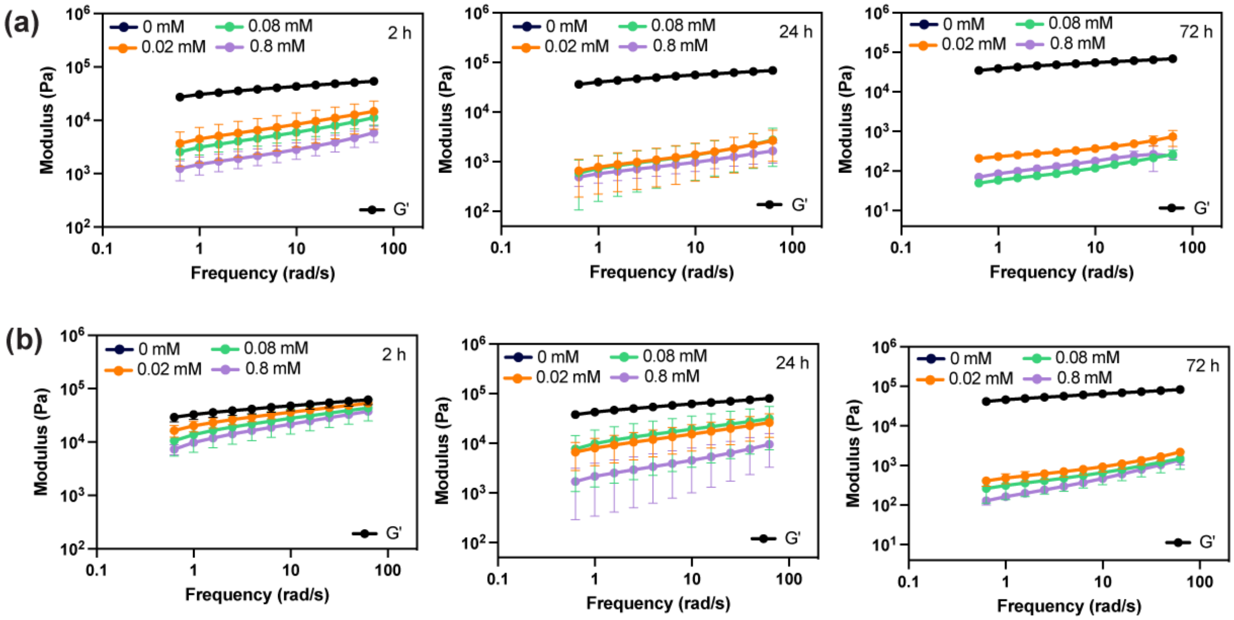

Figure S5. Frequency sweeps of the storage modulus for the PDADMAC/CMC complexes prepared at a 47.5/52.5 mol% PDADMAC/CMC mixing ratio and 3 mM total polymer in pH 7.0 buffer. The top row **(a)** shows the sPDADMAC/CMC system, and the bottom row **(b)** shows the lPDADMAC/CMC system. For each row, panels from left to right correspond to measurements collected 2 h, 24 h, and 72 h after cellulase addition. Samples contained 0 mM (black), 0.02 mM (orange), 0.08 mM (green), or 0.8 mM (purple) cellulase. Closed circles denote storage modulus G', and error bars represent the standard deviation of triplicate runs.

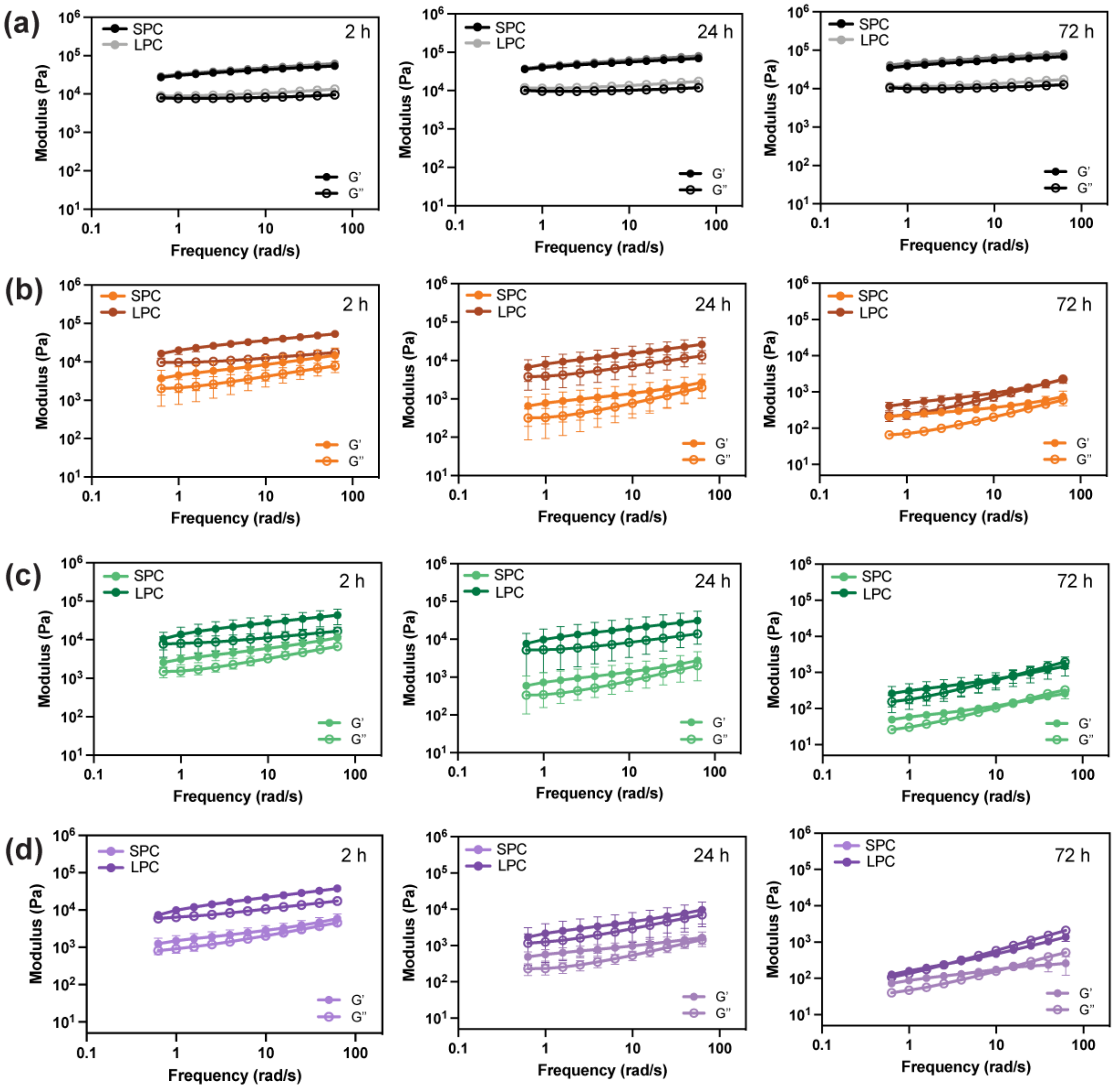

Figure S6. Frequency sweeps comparing sPDADMAC/CMC (SPC) and lPDADMAC/CMC (LPC) at 3 mM total polymer and 47.5/52.5 mol% PDADMAC/CMC, pH 7.0. Rows show cellulase concentrations of **(a)** 0 mM, **(b)** 0.02, **(c)** 0.08 mM, and **(d)** 0.8 mM. In each row, the three plots are 2 h, 24 h, and 72 h from left to right. SPC and LPC are plotted together for comparison at matched conditions. Error bars indicate the standard deviation of triplicate runs.

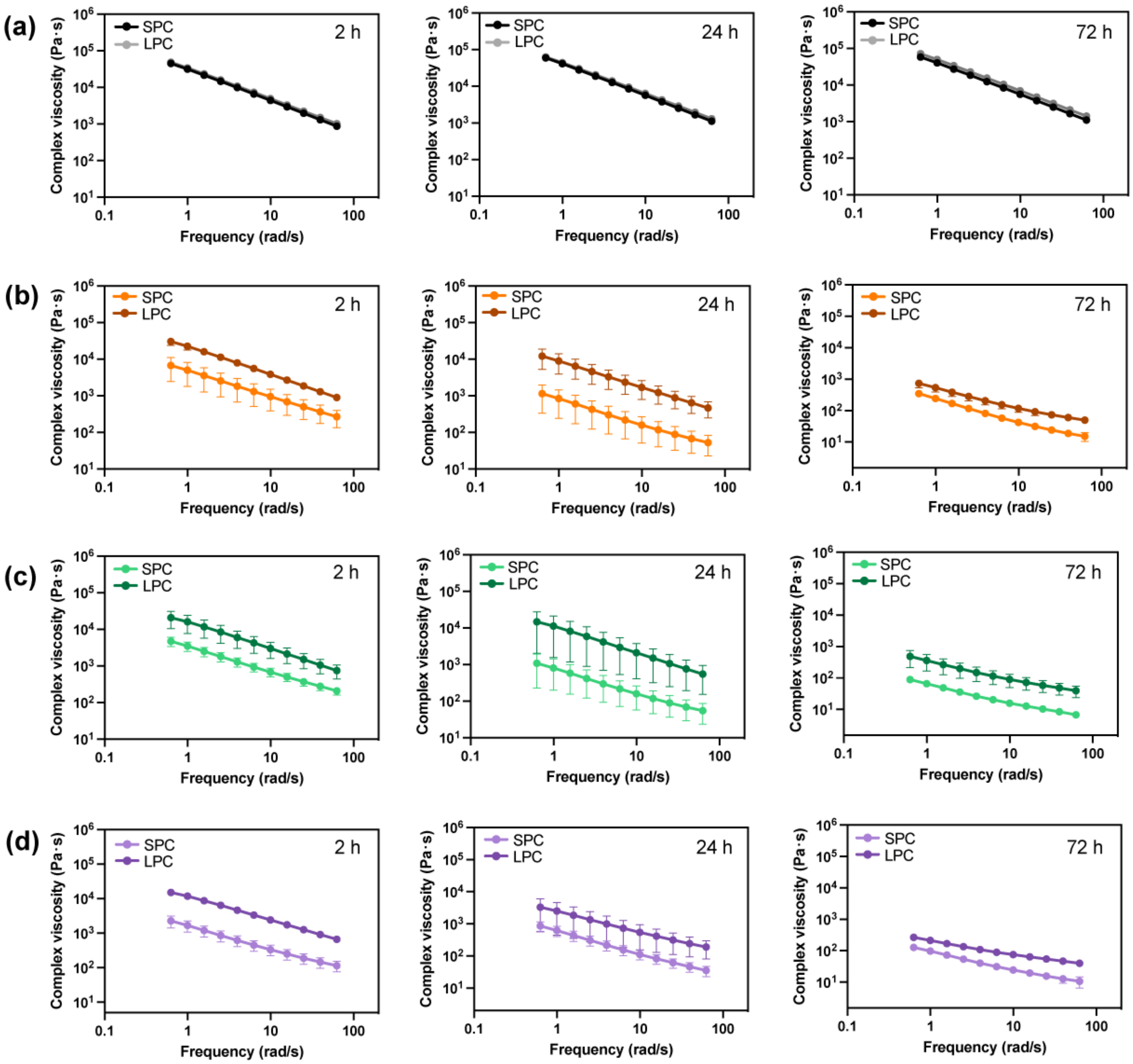

Figure S7. Complex viscosity comparing sPDADMAC/CMC (SPC) and lPDADMAC/CMC (LPC) at 3 mM total polymer and 47.5/52.5 mol% PDADMAC/CMC, pH 7.0. Rows show cellulase concentrations of **(a)** 0 mM, **(b)** 0.02, **(c)** 0.08 mM, and **(d)** 0.8 mM. In each row, the three plots are 2 h, 24 h, and 72 h from left to right. SPC and LPC are plotted together for comparison at matched conditions. Error bars indicate the standard deviation of triplicate runs.

References

1. H.M. Berman, J. Westbrook, Z. Feng, G. Gilliland, T.N. Bhat, H. Weissig, I.N. Shindyalov, P.E. Bourne, The Protein Data Bank (2000) *Nucleic Acids Research* 28: 235-242 https://doi.org/10.1093/nar/28.1.235.

MATLAB Script for Charge Calculations

```
function AASeq_Charge_Calc

% Amino Acid Sequence Charge Calculator by Whitney Blocher McTigue

% Amino acid information taken from pp 1086-88 of Organic Chemistry by T.W.
% Graham Solomons and Craig B. Fryhle, 10th edition

% Calulations based on Henderson-Hasselbalch equation and Moore, 'Amino
% Acid and Peptide Net Charges: A simple Calculational Procedure,'
% Biochemical Education, 13(1), 1985.

clear, clc

seq = input('AA Sequence as a String:\n'); % Sequence input
% This input needs to have single quotes around it.
pH = input('pH Value or Range:\n'); % pH range input

n = length(seq); % Determines the length of the sequence

% Charged AA
D = count(seq, 'D'); % Aspartate (Asp)
E = count(seq, 'E'); % Glutamate (Glu)
K = count(seq, 'K'); % Lysine (Lys)
R = count(seq, 'R'); % Arginine (Arg)
H = count(seq, 'H'); % Histidine (His)

% Other AA
Y = count(seq, 'Y'); % Tryosine (Tyr)
C = count(seq, 'C'); % Cysteine (Cys)
G = count(seq, 'G'); % Glycine (Gly)
A = count(seq, 'A'); % Alanine (Ala)
V = count(seq, 'V'); % Valine (Val)
L = count(seq, 'L'); % Leucine (Leu)
I = count(seq, 'I'); % Isoleucine (Ile)
M = count(seq, 'M'); % Methionine (Met)
F = count(seq, 'F'); % Phenylalanine (Phe)
W = count(seq, 'W'); % Tryptophan (Trp)
P = count(seq, 'P'); % Proline (Pro)
S = count(seq, 'S'); % Serine (Ser)
T = count(seq, 'T'); % Threonine (Thr)
N = count(seq, 'N'); % Asparagine (Asn)
```

```
Q = count(seq, 'Q'); % Glutamine (Gln)

MW_G = G.*57.052; % in g/mol
MW_A = A.*71.079;
MW_V = V.*99.133;
MW_L = L.*113.160;
MW_I = I.*113.160;
MW_M = M.*131.199;
MW_F = F.*147.177;
MW_W = W.*186.214;
MW_P = P.*97.117;
MW_S = S.*87.078;
MW_T = T.*101.105;
MW_C = C.*103.145;
MW_Y = Y.*163.176;
MW_N = N.*114.104;
MW_Q = Q.*128.131;
MW_D = D.*115.088;
MW_E = E.*129.115;
MW_K = K.*128.175;
MW_R = R.*156.189;
MW_H = H.*137.142;

MW = 18.015 + MW_G + MW_A + MW_V + MW_L + MW_I + MW_M + MW_F + MW_W + MW_P...
    + MW_S + MW_T + MW_C + MW_Y + MW_N + MW_Q + MW_D + MW_E + MW_K + MW_R...
    + MW_H; % in g/mol, 18.015 adds H- to N terminous and -OH to C terminous

Nterm = seq(1); % The N-terminus
Cterm = seq(n); % The C-terminus

% Determines what the N-terminus is and lists the pKa.
if Nterm == 'A'
    NT = 9.7;
elseif Nterm == 'R'
    NT = 9.0;
elseif Nterm == 'N'
    NT = 8.8;
elseif Nterm == 'D'
    NT = 9.8;
elseif Nterm == 'C'
    NT = 10.8;
elseif Nterm == 'Q'
    NT = 9.1;
elseif Nterm == 'E'
    NT = 9.7;
elseif Nterm == 'G'
    NT = 9.6;
elseif Nterm == 'H'
    NT = 9.2;
elseif Nterm == 'I'
    NT = 9.7;
elseif Nterm == 'L'
    NT = 9.6;
elseif Nterm == 'K'
    NT = 9.0;
elseif Nterm == 'M'
```

```
    NT = 9.2;
elseif Nterm == 'F'
    NT = 9.1;
elseif Nterm == 'P'
    NT = 10.6;
elseif Nterm == 'S'
    NT = 9.2;
elseif Nterm == 'T'
    NT = 10.4;
elseif Nterm == 'W'
    NT = 9.4;
elseif Nterm == 'Y'
    NT = 9.1;
elseif Nterm == 'V'
    NT = 9.6;
end

% Determines what the C-terminus is and lists the pKa.
if Cterm == 'A'
    CT = 2.3;
elseif Cterm == 'R'
    CT = 2.2;
elseif Cterm == 'N'
    CT = 2.0;
elseif Cterm == 'D'
    CT = 2.1;
elseif Cterm == 'C'
    CT = 1.7;
elseif Cterm == 'Q'
    CT = 2.2;
elseif Cterm == 'E'
    CT = 2.1;
elseif Cterm == 'G'
    CT = 2.3;
elseif Cterm == 'H'
    CT = 1.8;
elseif Cterm == 'I'
    CT = 2.4;
elseif Cterm == 'L'
    CT = 2.4;
elseif Cterm == 'K'
    CT = 2.2;
elseif Cterm == 'M'
    CT = 2.3;
elseif Cterm == 'F'
    CT = 1.8;
elseif Cterm == 'P'
    CT = 2.0;
elseif Cterm == 'S'
    CT = 2.2;
elseif Cterm == 'T'
    CT = 2.6;
elseif Cterm == 'W'
    CT = 2.4;
elseif Cterm == 'Y'
    CT = 2.2;
```

```
elseif Cterm == 'V'
    CT = 2.3;
end

% Calculates the charge at each pH value
Q = 1./(1+10.^(pH-NT))  - 1./(1+10.^(-(pH-CT)))  - D./(1+10.^(-(pH-3.9)))...
    - E./(1+10.^(-(pH-4.3)))  + K./(1+10.^(pH-10.5))...
    + R./(1+10.^(pH-12.5))  + H./(1+10.^(pH-6.0));  %- Y./(1+10.^(-(pH-10.1)));%...
    %- C./(1+10.^(-(pH-8.3)));

% Calculations for pI value
pH_pI = 0.1:0.001:14; % pH range for the calculation of pI

Q_pI = 1./(1+10.^(pH_pI-NT))  - 1./(1+10.^(-(pH_pI-CT)))  - D./(1+10.^(-(pH_pI-3.9)))...
    - E./(1+10.^(-(pH_pI-4.3)))  + K./(1+10.^(pH_pI-10.5))...
    + R./(1+10.^(pH_pI-12.5))  + H./(1+10.^(pH_pI-6.0));  %- Y./(1+10.^(-(pH_pI-10.1)))...
%    - C./(1+10.^(-(pH_pI-8.3)));  % Charge calculation for each pH point

ub = find(Q_pI<0,  1, 'first');  % When the charge first becomes positive
lb = find(Q_pI>0,  1, 'last'); % When the charge is last to be negative
%
ub2 = find(Q_pI<-25.5/2,  1, 'first'); % When the charge first becomes positive
lb2 = find(Q_pI>-25.5,  1, 'last'); % When the charge is last to be negative
%     % Finds the upper and lower bounds of the sequence where it most closely
%     % matches 0.

pI = 0.5.*(0.5.*(2.*pH_pI(lb)-1)  + 0.5.*(2.*pH_pI(ub)+1));
    % Uses the upper and lower bounds to then calculate the pI.

pKa = 0.5.*(0.5.*(2.*pH_pI(lb2)-1)  + 0.5.*(2.*pH_pI(ub2)+1));

pos_Q = K./(1+10.^(pH-10.5))  + R./(1+10.^(pH-12.5))  + H./(1+10.^(pH-6.0))  + 1./(1+10.^(pH-NT));
    % Calculates number of positive charges

neg_Q =  D./(1+10.^(-(pH-3.9)))  + E./(1+10.^(-(pH-4.3)))  + 1./(1+10.^(-(pH-CT)));
    % Calculates number of negative charges
ratio_Q = neg_Q./pos_Q;
Abs_Q = neg_Q  + pos_Q;
figure(1)
plot(pH_pI,  Q_pI,'LineWidth',1)
axis([0,14,-inf,inf])
xlabel('pH','fontsize',10,'fontweight','bold','FontName','Arial')
ylabel('Net  Charge','fontsize',10,'fontweight','bold','FontName','Arial')
set(gca,'YMinorTick','on','XMinorTick','on','LineWidth',1)
axis square
title(['Charge  vs.  pH for  ' input('name  of sequence\n')])
hold on
plot(pI,  0,  'k*','MarkerSize',8)  %,pKa,0.5,'ro'
%plot(pI_exp,0,  'rd','MarkerSize',8)
hold off
% fig_name  = input('Filename:\n');

% set(figure(1),  'PaperUnits',  'centimeters','PaperPosition',[0   0 17.1  10]);
% print(figure(1),   '-depsc',  fig_name,'-r600');

% data_out = [pH_pI',  Q_pI'];
```

```
% file_name = input('File name:\n');
% % xlswrite(file_name,data_out)

% table = [pH; Q; pos_Q; neg_Q; ratio_Q; Abs_Q; n];
% fprintf('pI = %0.2f\n',pI)
% % fprintf('pKa = %0.2f\n',pKa)
% %fprintf('pKa = %0.2f\n',pKa)
% fprintf('pH\t\tQ\t\tPos Q\t\tNeg Q\t\tN/P Ratio\t Abs Q\tResidues\n')
% fprintf('%0.1f\t\t%0.2f\t\t%0.2f\t\t%0.2f\t\t%0.2f\t\t%0.2f\t%0.2f\n',table)
% fprintf('Molecular Weight = %0.6f kDa\n', MW/1000)
```
